# Combined Use of Savi Scout and Magtrace for Non-Palpable Breast Cancer: A Retrospective Study

**DOI:** 10.3390/cancers18193197

**Published:** 2026-10-03

**Authors:** Claire Lloyd-Davies, Abdul Kasem

**Affiliations:** 1 London North West University Healthcare NHS Trust, London HA1 3UJ, UK; 2 King’s College Hospital NHS Foundation Trust, London BR6 8ND, UK; 3 HCA Healthcare London Bridge Hospital, London SE1 2PR, UK

**Keywords:** breast-conserving surgery, breast neoplasm, non-palpable breast cancer, sentinel lymph node biopsy, tumor localization

## Abstract

Background: Non-palpable breast cancer management has become less invasive and more efficient, driven by advances in tumor localization and sentinel lymph node (SLN) identification. Savi Scout, using a radar-based localization system, can be implanted into the tumor before neoadjuvant systemic therapy (NST). Magtrace, a superparamagnetic iron oxide nanoparticle for SLN detection, can be administered preoperatively. This study evaluated combining Savi Scout and Magtrace for breast-conserving surgery and SLN biopsy in non-palpable breast cancer. Methods: Patients who underwent breast-conserving surgery and SLN biopsy for non-palpable breast cancer using Savi Scout and Magtrace between December 2022 and June 2026 at HCA Healthcare London Bridge Hospital were retrospectively identified. Outcomes included successful tumor localization, SLN identification, intraoperative signal detection failure, margin status, re-excision rate and staining. Results: 72 patients were identified (mean age 52 years, standard deviation ± 10), with a total of 78 breast lesions and 73 SLN biopsies. A total of 27.8% (20/72) underwent neo-adjuvant therapy with Savi Scout in situ. SLNs were identified using Magtrace in 95.9% (70/73) of SLN biopsies, with a mean of 2.7 (standard deviation ± 0.7) SLNs identified per procedure. A total of 78 Savi Scout devices were implanted, one for each lesion. Of these, 97.4% (76/78) were localizable intraoperatively, 100.0% (78/78) were retrieved and none migrated. One patient required re-excision for margin involvement. Conclusions: Combined use of Savi Scout and Magtrace is feasible, demonstrates high technical success, and offers pre-operative imaging flexibility in primary surgery for non-palpable breast cancers and post-NST patients. This study evaluates the combination of Savi Scout and Magtrace and demonstrates a practical approach for simultaneous tumor localization and SLN identification.

## Simple Summary

Accurately marking breast cancers and sentinel lymph nodes is vital for successful cancer treatment, especially when the cancers cannot be felt (non-palpable). Traditionally, this involved inserting a wire before the operation, but increasingly less invasive techniques have been developed that also allow scheduling flexibility and can be used alongside cancer treatments such as chemotherapy. In this study, we evaluate the combined use of two such techniques in 72 patients who underwent breast-conserving surgery with sentinel lymph node biopsy. We evaluated Savi Scout to localize non-palpable breast cancers and Magtrace to identify the sentinel lymph node. We found this combination to be feasible and highly successful, offering flexibility in pre-operative imaging and scheduling.

## 1. Introduction

Breast cancer is the most common cancer in women in the UK and accounts for 30% of all new cancer cases in women [1]. Increased uptake and advances in the UK breast screening program identified 32% of breast cancer cases diagnosed in England in 2019 [2]. With increasing numbers of cancers being diagnosed through screening, there has been a rise in detection of non-palpable breast cancer.

Several non-palpable tumor localization and sentinel lymph node (SLN) identification techniques exist, with recent developments becoming increasingly less invasive for patients. These include wire-guided localization (WGL), which remains widely used, and radioactive tracers or localizers [3,4,5,6]. More recently, novel wire-free techniques that do not use radioactive material have increased in popularity, such as Savi Scout (a radar-based localization system), Magseed (magnetic seeds) and radiofrequency identification (RFID) tags [3,7,8,9,10,11].

Previously, the SLN may have been identified using Patent Blue V dye and Technetium-99 radioisotopes, or a combination of both. However, issues such as skin staining, blind dissection, anaphylaxis and the requirement for nuclear medicine facilities resulted in the development of Super Paramagnetic Iron Oxide (SPIO) particles, such as Magtrace [12,13,14,15].

Non-palpable tumor localization and SLN identification may be combined. Magtotal, a combination of Magtrace and Magseed, is one such example, which, although feasible, has challenges arising from overlapping magnetic signals [16,17]. Another notable issue with Magseed is that its MRI artifact can measure 4–6 cm due to the iron content, which may limit diagnostic accuracy of MRI whilst the seed is in place [7,18]. Therefore, clinicians may prefer to use a localizer such as Savi Scout, which has minimal artifact [7,18].

This retrospective study evaluated the combination of Savi Scout and Magtrace for breast-conserving surgery and SLN biopsy in non-palpable breast cancer. It assesses whether this combined approach is feasible to localize the tumor and identify the SLN, and whether it may address any of the recognized limitations of the alternative combinations discussed above.

## 2. Materials and Methods

Patient records from HCA Healthcare London Bridge Hospital were screened between December 2022, when the site began using Savi Scout and Magtrace in combination, and June 2026. Patients who underwent breast-conserving surgery with SLN biopsy using a combination of Savi Scout and Magtrace were identified and consecutively included.

### 2.1. Protocol

Savi Scout and Magtrace are administered at the same appointment within seven days of the operation, unless the patient is undergoing neoadjuvant systemic therapy (NST). If NST is indicated, Savi Scout is inserted prior to starting NST, and Magtrace is injected after NST completion and within seven days of the operation. The standard oncology NST protocol is 6 months in duration, with the Savi Scout device implanted prior to commencing NST. All operations are completed within a range of 3–6 weeks post-NST completion. In the department, two breast radiologists consistently completed Savi Scout and Magtrace administration.

Savi Scout is implanted under ultrasound guidance into the center of the lesion, at a depth determined by the lesion location. If a patient has more than one lesion, one device is implanted into each. Placement is confirmed using the Scout Check Console and a mammogram. Magtrace is then injected and the Savi Scout signal is reconfirmed. If the Savi Scout signal is weak or not detectible, WGL is performed within 24 h of the operation to provide an alternative in case the Savi Scout signal is not detectable intraoperatively.

1 mL of Magtrace is injected under local anesthetic into the tumor margins (peritumoral injection), superior-lateral to the Savi Scout. The depth of injection is dictated by tumor depth and is above the fascial plane of pectoralis. Injection is completed using ultrasound guidance because free-handed, non-radiology-guided injection is a predictor for post-operative MRI artifacts in peritumoral injection [19]. The Magtrace volume of 1 mL has been demonstrated as non-inferior to higher doses and is associated with significantly less skin staining [20,21,22].

Administration of Magtrace pre-operatively confers scheduling flexibility, shortens operative time and has been demonstrated to improve SLN identification [19,20,22]. Preoperative injection of SPIO increases the number of SLNs identified, without tracer spillage or diminished SLN visualization [19,22]. Removing the requirement for peri-operative Magtrace injection allows scheduling flexibility and decoupling of the surgical and radiological procedures. It shortens operative time, as there is no requirement for the peri-operative injection and breast massage has not been shown to be necessary [20,22].

Post-injection breast massage is not completed at the institution, as observed in other protocols using pre-operative peritumoral SPIO injection [19,20,22]. It has not been shown to be necessary for tracer migration and SLN identification in pre-operative peritumoral or retroareolar injection [22]. Furthermore, a meta-analysis found the lowest discoloration rates were associated with lower SPIO dose, peritumoral injection, and pre-operative injection without massage [20].

Peritumoral administration allows removal of the SPIO during breast-conserving surgery, which addresses MRI artifact concerns on post-operative imaging and reduces the frequency of skin staining [19,22]. Furthermore, a meta-analysis comparing SPIO to radioisotopes with and without blue dye found peritumoral injection of SPIO was associated with better SLN detection [20].

All operations were completed by the same surgeon. On the day of operation, the signal from both Savi Scout and Magtrace are confirmed prior to commencing pre-anesthetic administration and re-confirmed intraoperatively. Savi Scout location is confirmed using the Scout Check console, and the site is also marked. After excision of the tumor, Savi Scout retrieval is confirmed by X-ray of the specimen prior to completion of the operation and later reconfirmed within the histopathology specimen. The X-ray is checked by the operating surgeon intraoperatively and reported post-operatively by a radiologist. The surgical excision specimen is also examined intraoperatively; the presence of the Magtrace within the specimen is confirmed visually by staining and by the presence of signal using the Sentimag probe. Staining within the specimen is later reconfirmed on the histology specimen. Removal of Magtrace footprint from the operation site after SLN excision is verified by examining the wound bed for any remnant staining and using the Sentimag probe to identify residual Magtrace signal. If Magtrace is not detectible intraoperatively, Patent Blue V dye is used as an alternative on the day. Although a radioisotope is available within the institution, due to the associated requirement of nuclear medicine, Patent Blue V dye is more readily available and therefore used as an alternative if Magtrace fails to identify the SLN on the day of operation.

### 2.2. Participants

#### 2.2.1. Inclusion Criteria

- Adult patients (≥18 years).
- Non-palpable breast lesion and axillary lymph node identified using Savi Scout and Magtrace, respectively.

#### 2.2.2. Exclusion Criteria

- Nickel allergy.
- Pacemaker or implanted device in chest wall.
- Pregnancy or lactation.
- Palpable breast lesion.
- Hypersensitivity to iron oxide or dextran compounds.
- Iron overload disease.

### 2.3. Data Collection

We reviewed patient data at HCA Healthcare London Bridge Hospital, including clinical records, surgical operation notes, radiology reports, post-operative histopathology and breast cancer multidisciplinary team meeting outcomes. We collected patient demographic information, including age at surgery and location of lesion.

Primary outcomes included successful localization rate of the non-palpable tumors intraoperatively using Savi Scout and successful identification of the SLN using Magtrace. Successful localization rate using Savi Scout was defined as intraoperative identification and is reported as a proportion of the total number of Savi Scout devices. Successful identification of the SLN using Magtrace is reported as a proportion of the total number of SLN biopsy procedures.

Secondary outcomes included pre-operative Savi Scout device signal strength, Savi Scout device migration, intraoperative Savi Scout device or Magtrace signal detection failure, intraoperative complications, interference events, Savi Scout device retrieval, margin status, re-excision rate, post-operative complications, and skin staining. Savi Scout device migration was defined as movement of the device from its pre-operative intralesional position to outside of the lesion. Device placement pre- and post-operatively was assessed as outlined within the protocol (Section 2.1). Interference events were defined as intraoperative difficulty locating a Savi Scout device signal and are reported as a proportion of the total number of devices. A negative margin is locally defined as a 1 mm free margin from cancer, consistent with the Association of Breast Surgery’s guidance, and is determined in combination with the histopathology report and MDT consensus [23].

Nodal status post-NST was assessed by examining axillary lymph nodes using US and contrast-enhanced breast MRI scan. All included NST patients were discussed at breast MDT post-NST treatment, where breast-conserving surgery was deemed an appropriate management option. For NST patients, pathological response was collected as defined by the post-operative histopathology report and breast cancer MDT decision. Partial pathological response is defined as the presence of residual tumor, and complete pathological response is defined by the absence of any residual tumor. Radiological response was not assessed as the efficacy and response to NST was not the focus of this study.

### 2.4. Statistical Analysis

Descriptive statistics were used to summarize patient, tumor and outcome data. Categorical variables are reported as whole numbers with percentages. The 95% confidence intervals (CIs) for primary and secondary outcomes were calculated using the Wilson score method.

Continuous variables were assessed for normality using the Shapiro–Wilk test. Continuous variables are reported as mean and standard deviation (SD). Age at surgery, specimen weight and number of SLN identified were compared between patients who did and did not receive NST using the Mann–Whitney U test. Specimen weight was treated as per-lesion, so lesions from the same patient were included independently.

## 3. Results

### 3.1. Cohort

**Figure 1:**
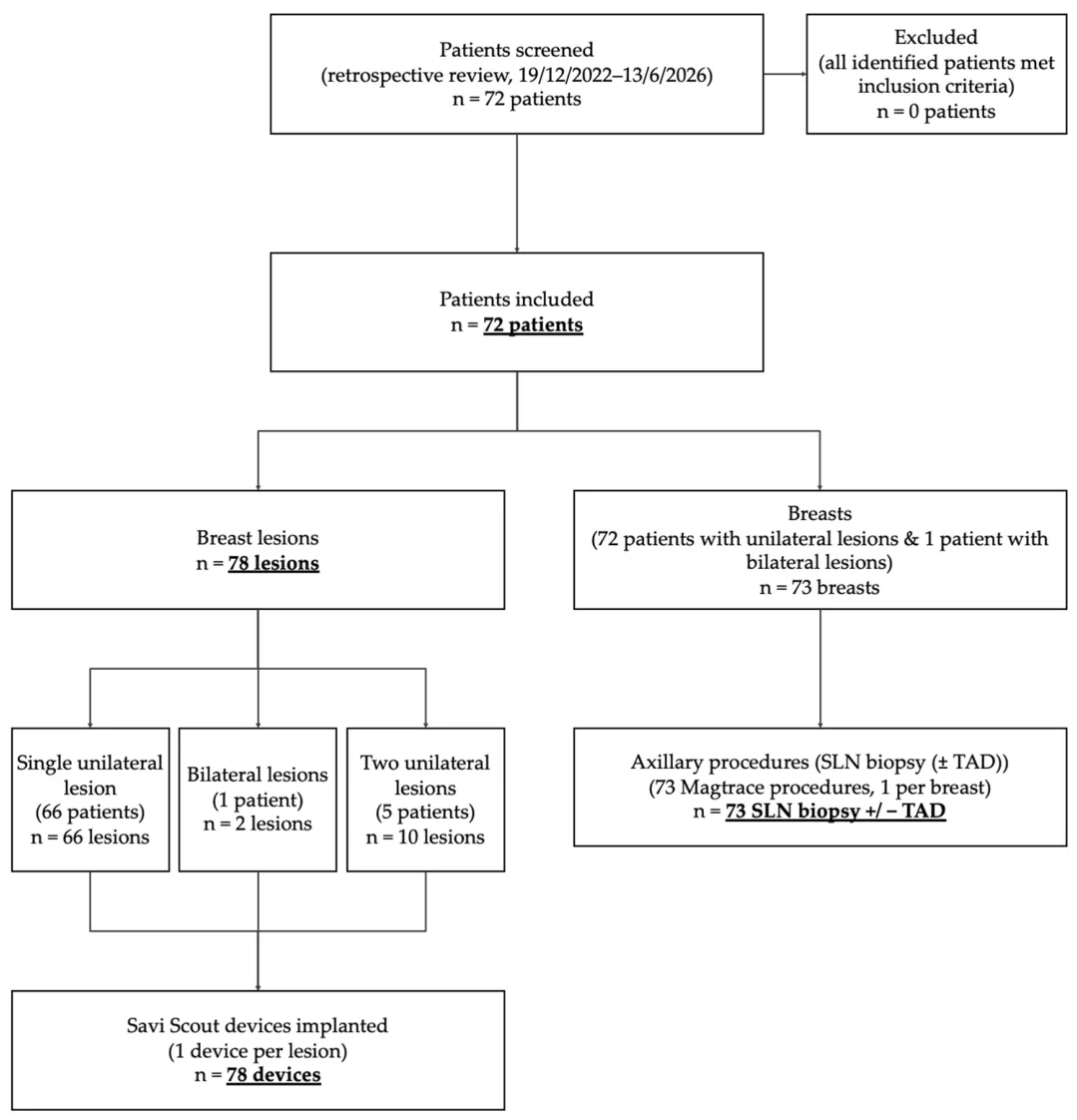
Study flow diagram detailing the cohort information. Overall, there were 72 patients with 78 lesions and 73 axillary procedures completed. TAD = Targeted Axillary Dissection.

72 patients underwent surgery and SLN biopsy using a combination of Savi Scout and Magtrace between 19 December 2022 and 13 June 2026. Figure 1 details the number of patients, lesions, Savi Scout devices implanted, and breast and axillary procedures completed. No patients were excluded; all met inclusion criteria.

Two patients (2.8%, 2/72) had a history of prior breast surgery involving implants: one following a bilateral mastectomy with implant reconstruction, and another following cosmetic breast augmentation. The patient with a prior bilateral mastectomy had a local recurrence and underwent a wide local excision with SLN biopsy; they were included in this study as the breast implant was conserved.

Table 1 details patient characteristics. The mean age was 52 years.

Across all tumors included within the study cohort, the most frequent histology reported was No Special Type (79.5%, 62/78) (Table 1). For invasive cancers, grade 2 histology was the most frequently observed grade (65.4%, 51/78), followed by grade 3 (26.9%, 21/78) and grade 1 (6.4%, 5/78).

### 3.2. SLN Identification and Tumor Localization

The mean number of SLNs identified per procedure was 2.7 (SD ± 0.7, range 1–4), including those who also had Targeted Axillary Dissection (TAD) (Table 2).

SLNs were successfully identified using Magtrace in 95.9% (70/73) of procedures (Table 2). The denominator of 73 reflects 72 patients with unilateral breast lesions and one patient with bilateral breast lesions who contributed two independent axillary procedures (Figure 1). Magtrace did not identify a SLN and Patent Blue V dye was injected as an alternative in three procedures (4.1%, 3/73). In two of these procedures, there was no blue dye uptake, and so four lymph nodes were sampled in both. Of the two patients where both Magtrace and blue dye uptake failed, one had a high BMI (BMI > 30). These two patients had a mean age of 47, no previous ipsilateral axillary surgery and had not received axillary or chest wall radiation. In the remaining patient, blue dye identified the SLN and two were removed. This patient did not have a high BMI, had no previous ipsilateral axillary surgery, and had not received axillary or chest wall radiotherapy.

Savi Scout devices were implanted into every breast lesion. Where a patient had two or more lesions, a device was implanted in each lesion. Overall, 78 Savi Scout devices were implanted, with 66 implanted into 66 patients with single tumors and 12 implanted into six patients with two tumors each (Figure 1). At the pre-operative review, 96.2% (75/78) had a strong signal, 1.3% (1/78) had a weak signal, and 2.6% (2/78) were not detectible (Table 2). The 2.6% that were not detectible occurred early following the introduction of the technique to the institution. WGL was used as a localizing alternative for the three patients with weak or not detectible signals. Intraoperatively, 97.4% (76/78) of devices were localizable, including the device with a pre-operative weak signal (Table 2). The remaining two Scout devices (2.6%, 2/78) that were not detectable pre-operatively could not be localized intraoperatively, and so the previously implanted WGL was used. The causes of Savi Scout signal loss were not established. All devices (100.0%, 78/78) were successfully retrieved on tumor excision and no devices migrated (0.0%, 0/78) (Table 2). There were no observed intraoperative interference events (0.0%, 0/78) (Table 2). The denominator was set at 78 as the events were reported as a proportion of the total number of Savi Scout devices.

### 3.3. Patient Outcomes

Clear resection margins were achieved in 98.7% (77/78) of tumors (Table 2). A single patient had involved margins (1.3%, 1/78, 95% CI 0.2–6.9%) and therefore underwent re-excision, with a consequent re-excision rate of 1.4% (1/72). No other patients required re-excision.

The operating time ranged from 70 to 120 min. All patients stayed for a duration of 1 day.

Table 2 details complications. No patients had intraoperative complications (0.0%, 0/72); 4.2% (3/72) developed infected seromas, which resolved with antibiotics and aspiration; 2.8% (2/72) experienced delayed healing associated with infected seromas; 1.4% (1/72) developed a post-operative axilla hematoma which required return to theater for surgical evacuation.

A total of 2.8% (2/72) of patients experienced skin staining overlying the tumor postoperatively, all <2 cm. Both affected patients remain under follow-up, with ongoing gradual resolution of staining.

All patients underwent annual post-operative follow-up, which includes routine mammography with or without ultrasound as required. Annual follow-up continues for 5 years, or until the patient reaches age 50, whichever is later. No patients required breast MRI within 1 year post-operatively, whether for follow-up or other indications such as new clinical findings concerning for recurrence.

### 3.4. NST Subgroup

Twenty patients (20/72, 27.8%) underwent neo-adjuvant therapy prior to their surgery, with Savi Scout in situ. NST achieved a complete pathological response in eleven tumors and a partial response in a further eleven tumors (Table 3). A total of 100.0% (20/20) of NST patients had tumors that were localized by Savi Scout and 100.0% (20/20) had SLN identified by Magtrace. Table 3 details further NST cohort information.

Age, specimen weight and number of SLN identified did not differ significantly between patients who received NST and those who did not. Age was normally distributed in the NST group, but not the non-NST group (Shapiro–Wilk *p* = 0.058 and *p* = 0.005, respectively). Specimen weight and SLN were not normally distributed in either group (Shapiro–Wilk *p* < 0.001 for all). Mann–Whitney U demonstrated no significant differences between the NST group and non-NST group for age (U = 450, *p* = 0.378), specimen weight (U = 491, *p* = 0.267), or number of SLN identified (U = 455, *p* = 0.303).

As per local protocol, all patients with confirmed three metastatic lymph nodes or less at diagnosis, who have completed NST, are eligible for TAD. Normalization of the previously sampled metastatic lymph node(s) on repeat imaging allows their removal using TAD, rather than complete axillary lymph node dissection. The PET CT and histopathology sampling for all patients are discussed in the breast MDT and TAD is recommended for these patients. The positive node is clipped before starting neoadjuvant therapy. TAD is then completed, in addition to SLN biopsy, to remove the clipped lymph node which was previously identified as having metastatic disease. A total of 25.0% (5/20) of the patients who received NST had a clinical nodal status of N1 at the time of diagnosis, before commencing NST. After finishing NST, the axillary lymph nodes of these five patients appeared normalized on repeat imaging. Consequently, these patients underwent TAD in addition to breast-conserving surgery and SLN biopsy (Table 3). The remaining 75% (15/20) of patients had normal (N0) appearing lymph nodes on imaging at the time of diagnosis, before commencing NST, and again on repeat imaging after NST completion. These patients underwent SLN biopsy without TAD.

## 4. Discussion

Our study of 72 patients assesses whether the combined use of Magtrace and Savi Scout is practical to localize the tumor and identify the SLN.

### 4.1. Interpretation of Findings

The high rate of SLN detection demonstrates that lower volume and peritumoral Magtrace injection is compatible with Savi Scout use. We were able to reliably detect 95.9% of SLNs using Magtrace, which is in line with the 89.7–100.0% detection rates reported in the literature [24,25]. In the remaining three patients, Patent Blue V dye was injected as an alternative, which may be used as part of a dual tracer technique if required [16]. Of note, two of these patients had no uptake with Patent Blue V dye. One of these patients had a high BMI, which is recognized to reduce the likelihood of successful SLN mapping [26]. Their mean age (47 years) was not greater than the overall cohort mean (52 years), and neither had previous ipsilateral axillary surgery or radiotherapy [26,27].

A systematic review and pooled analysis of 842 cases completed by Kasem and Mokbel demonstrated Savi Scout to be a safe and effective alternative to WGL, with a successful retrieval rate of 99.64% [8]. Our 97.4% rate of successful localization using Savi Scout and 100.0% retrieval rate are consistent with the literature [8,28]. Furthermore, Savi Scout was successfully retrieved in a range of clinical scenarios, including post-NST, after TAD, following previous mastectomy, and in the presence of breast implants. Although these subgroups were small, these initial findings suggest feasibility, warranting further exploration in future studies.

Savi Scout devices are recognized to experience detection difficulties or failure, and reported cases exist without concomitant use of Magtrace. Contributing factors can include mechanical damage or damage from electrocautery, the presence of post-biopsy hematomas or calcified fibroadenomas [29]. In our cohort, two Savi Scout devices could not be identified pre-operatively. These two devices subsequently could not be identified intra-operatively. The causes were not identified, although as the signal was initially confirmed and then lost immediately after Magtrace injection within the same appointment, and they were early in the use of this technology within the hospital, we propose this may be due to mechanical damage at the time of Magtrace injection [29]. In these patients, WGL was used as an alternative localizer. There were no further signal issues with Savi Scout devices. The presence of Savi Scout signal should therefore be confirmed at multiple points throughout the patient journey, including immediately post-insertion, post-Magtrace injection, pre-anesthetic administration and again on the table prior to commencing the operation. In the event of signal loss or difficulty, the team may employ alternative localization methods such as WGL.

The combined use of Magtotal is recognized to be associated with signal interference between Magtrace and Magseed [16,17]. Haaren et al. identified several protocol alterations that addressed interference, including ensuring a distance of 4 cm or more between the seed and tracer, a retro-areolar injection site for Magtrace and using non-iron instruments [16]. We experienced no intra-operative interference events with combined Savi Scout and Magtrace use. However, in the absence of a comparator group, these findings should be interpreted as feasibility rather than superiority and further comparative studies are required to determine whether this combination adequately addresses the signal interference problem. The impact of interference between Magtrace and Magseed is debated within the literature, with Hersi et al. reporting that it allows easier identification of the tumor, whilst Haaren et al. argue the interference can result in an undesirable larger excision volume [16,17]. Further investigation into the impact of Savi Scout and Magtrace on excision volume is required.

Margin status and therefore re-excision rates are influenced by several factors beyond the localization device. Factors can include age, multifocality, tumor size, grade, receptor status and presence of DCIS [30]. In the absence of a comparator group, we cannot attribute our 1.4% re-excision rate to the use of Savi Scout combined with Magtrace alone. Nevertheless, we have demonstrated our re-excision rate to not be in excess of existing literature using Savi Scout alone. Further comparative studies to demonstrate the superiority of the combination with Magtrace should be completed.

The role and impact of NST in breast-conserving surgery compared to mastectomy remains uncertain. Conflicting evidence in the literature may be attributed to heterogeneity and lack of contemporary data [31]. More recent evidence suggests breast-conserving surgery following NST is oncologically safe and associated with comparable or superior long-term outcomes compared to mastectomy [31,32,33]. Improved outcomes included higher disease-free survival, distant disease-free survival, overall survival, breast cancer-specific survival and reduced distant recurrence [31,32,33]. Mastectomy in patients undergoing NST was found to be a prognostic factor for poor outcomes [31]. Localization strategies, such as Savi Scout, can facilitate breast-conserving surgery for non-palpable breast lesions, including those who undergo NST [29]. As evidence on outcomes of breast-conserving surgery after NST continues to develop, the role of tumor localizers and SLN tracers in facilitating this procedure in wider cohorts should be investigated.

Compared to the 4–6 cm artifact observed in Magseed, Savi Scout exhibits minimal MRI artifact, which allows flexibility of pre-operative imaging with the localizer in situ [18,28]. Savi Scout can be implanted far in advance of surgery. Wazir et al. completed a systematic review on its use in NST patients and found the mean deployment of the Savi Scout device to surgery was 52 days (range 1–202) [29]. The minimal artifact and capacity for long-term implantation allow the Scout device to be implanted prior to commencing NST and to remain in situ during breast MRI imaging pre-operatively. This approach was used in our subgroup of patients who underwent NST (27.8% of overall cohort), who had Savi Scout implanted before starting NST. In this subgroup, the device can be deployed at the time of biopsy, which has been reported in the literature to ensure high surgical precision post-NST and remove the need for additional procedures, saving patient and clinical time as well as resources [7,29]. In contrast, Magseed can only be placed 30 days pre-operatively and its significant MRI artifact precludes its use in pre-operative NST patients [7,11].

Nevertheless, the problem of MRI artifacts on scans is not completely resolved in this protocol. Magtrace and other superparamagnetic tracers cause artifacts on MRI. Where a patient is undergoing NST, Savi Scout is inserted prior to starting NST. Magtrace is then injected nearer to the time of surgery, after NST completion, to ensure there is no breast MRI artifact during NST monitoring. In select patients, where the standard surveillance ultrasound and mammography are insufficient, post-operative MRIs may be required and Magtrace artifacts may impact these [34,35]. Although it is not known how long after injection the problem persists, Krischer et al. identified tracer residue artifacts in 76.5% of their cohort treated with breast-conserving surgery after 42 months of follow-up [36]. Current National Institute for Health and Care Excellence (NICE) guidance states Magtrace would not be advised for patients likely to need MRI within 3 months after SLN biopsy [24]. Should post-operative imaging be required and concerns exist around artifact, research suggests the MRI protocol may be modified, or alternative imaging such as mammography and contrast-enhanced mammography (CEM) may be appropriate [16,34,35,37,38]. In our cohort, routine post-operative imaging using mammography and ultrasound was sufficient.

The protocol aims to align with increasing evidence that reducing residual post-operative SPIO footprint will reduce skin staining and MRI artifact in selective patients where MRIs are required post-operatively [19,20,21,22]. Removal of the Magtrace footprint from the operation site is confirmed by examining the wound bed for remnant staining and using the Sentimag probe to check residual signal. Although no patients required post-operative breast MRIs, the protocol aims to facilitate one if required, and the proposed reduction in artifact is based on the current literature. Current evidence suggests radiology-guided injection and a lower tracer volume, such as 1 mL, into the immediate peritumoral area that will subsequently be excised reduces post-operative MRI artifact [19,20]. Using a lower volume of 1 mL has been shown to be non-inferior to higher doses and is associated with significantly less skin staining, which may be further improved by peritumoral injection [20,21,22]. Our skin staining rate of 2.7% compares favorably with pooled meta-analysis data, in which skin staining rates were 25.2% (95% CI 9.4–41.0) for 1 mL SPIO dose, 15.4% (95% CI 0–36.2) for peritumoral injection and 24.4% (95% CI 6.3–42.6) for no post-injection massage [20]. Intraoperatively, after removal of the SLN, the SPIO footprint is checked using the Sentimag probe and visual confirmation that Magtrace brown discoloration is absent. Confirming the absence of SPIO footprint has been shown to be predictive of the absence of MRI artifact [19].

### 4.2. Limitations

The main limitation of this study is the retrospective, single-center, single-surgeon design. All procedures were performed within one institution by the same surgeon and by two breast radiologists. Whilst this provides the advantage of procedural consistency and a standardized protocol, it does limit generalizability. The results may reflect local expertise and a mature multidisciplinary workflow, rather than the performance of the technique in broader practice.

Furthermore, there was no comparison with alternative localization techniques. As such, this work demonstrates the feasibility of the technique, and larger, prospective, multicenter studies comparing Savi Scout combined with Magtrace against Magseed combined with Magtrace are warranted.

The NST subgroup of patients was small (20 patients), limiting the statistical power of subgroup analysis. Findings relating to the use of Savi Scout and Magtrace in NST patients should be considered hypothesis-generating, and larger powered studies are needed to confirm broader feasibility in this specific population.

## 5. Conclusions

The combination of Savi Scout and Magtrace in breast-conserving surgery and SLN biopsy is feasible and demonstrates high technical success in this cohort. The combined use conveys the recognized benefits of both technologies, including removing the need for radioactive tracers or implants and allowing flexibility of pre-operative imaging with Savi Scout in situ for NST patients. With increasing evidence that breast-conserving surgery following NST improves patient outcomes, the effective use of tumor localizers to facilitate breast-conserving surgery in wider cohorts is vital. Our rates of SLN detection, tumor localization and re-excision were in line with the literature, demonstrating feasibility. However, superiority and comparative benefit over alternative combinations, such as Magseed combined with Magtrace, wire-guided localization or other non-radioactive localization techniques, was not directly assessed. Comparative studies with alternative combinations are therefore required to assess superiority. Furthermore, the impact on MRI interference in follow-up scans after SPIO particles, such as Magtrace, requires further assessment.

## Figures and Tables

**Table 1:** Details patient characteristics. ILC = Invasive Lobular Carcinoma; DCIS = Ductal Carcinoma in Situ.

Domain		% (*n/N*)
Age in years (mean +/− SD)		52 ± 10
Neo-adjuvant Therapy (number of patients)		27.8% (20/72)
Cancer location (number of patients)		
	Right	54.2% (39/72)
	Left	44.4% (32/72)
	Bilateral	1.4% (1/72)
Nodal Stage (number of SLN biopsy procedures) ^1^		
	N0	93.2% (68/73)
	N1	6.8% (5/73)
Histology Type ^2^ (number of tumors)		
	No Special Type	79.5% (62/78)
	ILC	15.4% (12/78)
	Mucinous Carcinoma	2.6% (2/78)
	Metaplastic tumor	1.3% (1/78)
	DCIS ^3^	1.3% (1/78)
Histology Grade ^4^ (number of tumors)		
	1	6.4% (5/78)
	2	65.4% (51/78)
	3	26.9% (21/78)
	High Grade (DCIS) ^3^	1.3% (1/78)

^1^ Nodal stage at diagnosis. ^2^ Of 72 patients, one patient had bilateral lesions, and five patients had two unilateral lesions. Total 78 tumors in the cohort. For the 20 patients who underwent NST, their histology type and grade in this table refer to the status before NST. ^3^ The primary lesion in this patient was invasive cancer (No Special Type), which was the indication for SLN biopsy. DCIS was the second lesion in this patient. ^4^ Grade refers to the Nottingham histologic grade (1–3) for No Special Type, ILC, mucinous carcinomas and metaplastic tumors. DCIS is graded separately by nuclear grade (low, intermediate and high).

**Table 2:** Details patient outcomes, including intraoperative, histopathology and post-operative outcomes.

Outcome		% (*n/N*) (95% CI)
Number of SLN (number of SLN biopsy procedures)		
	1	1.4% (1/73)
	2	31.5% (23/73)
	3	49.3% (36/73)
	4	11.0% (8/73)
	TAD + 2	4.1% (3/73)
	TAD + 3	2.7% (2/73)
Procedures with SLN identified (number of SLN biopsy procedures)		95.9% (70/73) (95% CI 88.6–98.6%)
Resection margin on final histology (number of tumors)		
	Negative margin	98.7% (77/78) (95% CI; 93.1–99.8%)
	Margin involved	1.3% (1/78) (95% CI 0.2–6.9%)
Savi Scout pre-operative signal strength (number of devices)		
	Strong	96.2% (75/78) (95% CI 89.3–98.7%)
	Weak	1.3% (1/78) (95% CI 0.2–6.9%)
	Not detectible	2.6% (2/78) (95% CI 0.7–8.9%)
Savi Scout devices localized intraoperatively (number of devices)		97.4% (76/78) (95% CI 91.1–99.3%)
Savi Scout devices retrieved (number of devices)		100.0% (78/78) (95% CI 95.3–100.0%)
Savi Scout devices migrated (number of devices)		0.0% (0/78) (95% CI 0.0–4.7%)
Interference events (number of devices)		0.0% (0/78) (95% CI 0.0–4.7%)
Complications		
	Intraoperative complication (number of patients)	0.0% (0/72) (95% CI 0.0–5.1%)
	Necrosis (number of patients)	0.0% (0/72) (95% CI 0.0–5.1%)
	Infected seromas (number of patients)	4.2% (3/72) (95% CI 1.4–11.5%)
	Delayed healing (number of patients)	2.8% (2/72) (95% CI 0.8–9.6%)
	Post-operative hematoma (number of patients)	1.4% (1/72) (95% CI 0.2–7.5%)
	Mortality secondary to surgery (number of patients)	0.0% (0/72) (95% CI 0.0–5.1%)
Skin staining (number of patients)		2.8% (2/72) (95% CI 0.8–9.6%)

**Table 3:** NST patient treatment, margin status and histopathology outcomes.

Domain		% (*n/N*)
Neo-adjuvant Therapy (number of patients)		
	Chemotherapy	95.0% (19/20)
	Endocrine Therapy and CDK4/6 inhibitor	5.0% (1/20)
TAD (number of patients)		25.0% (5/20)
Margin status (number of tumors) ^1^		
	Negative margin	100.0% (22/22)
	Margin involved	0.0% (0/22)
Histology Type prior to NST (number of tumors) ^2^		
	No Special Type	90.9% (20/22)
	Metaplastic tumor	4.5% (1/22)
	DCIS ^3^	4.5% (1/22)
Histology Grade prior to NST (number of tumors)		
	Grade 1	4.5% (1/22)
	Grade 2	31.8% (7/22)
	Grade 3	59.1% (13/22)
	DCIS High Grade	4.5% (1/22)
Response to NST (number of tumors)		
	Partial Response	50.0% (11/22)
	Complete Response	50.0% (11/22)

^1^ Two patients (2/20) had two tumors, making the total tumor burden for the NST subgroup 22. ^2^ Histology type and grade within this table refer to before NST. ^3^ As noted in Table 1, the primary lesion in this patient was invasive cancer (No Special Type), which was the indication for SLN biopsy. DCIS was the second lesion in this patient.

## Data Availability

Data available on request from the authors.

## Abbreviations

The following abbreviations are used in this manuscript:

SLN	Sentinel Lymph Node
NST	Neoadjuvant Systemic Therapy
WGL	Wire-Guided Localization
RFID	Radiofrequency Identification
SPIO	Super Paramagnetic Iron Oxide
MRI	Magnetic Resonance Imaging
MDT	Multidisciplinary Team
US	Ultrasound
SD	Standard Deviation
ILC	Invasive Lobular Carcinoma
DCIS	Ductal Carcinoma in Situ
TAD	Targeted Axillary Dissection
CI	Confidence Interval
BMI	Body Mass Index
NICE	National Institute for Health and Care Excellence
CEM	Contrast Enhanced Mammography
